# Presenting artificial intelligence, deep learning, and machine learning studies to clinicians and healthcare stakeholders: an introductory reference with a guideline and a Clinical AI Research (CAIR) checklist proposal

**DOI:** 10.1080/17453674.2021.1918389

**Published:** 2021-05-14

**Authors:** Jakub Olczak, John Pavlopoulos, Jasper Prijs, Frank F A Ijpma, Job N Doornberg, Claes Lundström, Joel Hedlund, Max Gordon

**Affiliations:** a Institute of Clinical Sciences, Danderyd University Hospital, Karolinska Institute, Sweden; b Department of Computer and System Sciences, Stockholm University, Sweden; c Flinders University, Adelaide, Australia; d Department of Trauma Surgery, University Medical Center Groningen, University of Groningen, Groningen, The Netherlands; e The Machine Learning Consortium; f Center for Medical Image Science and Visualization, Linköping University, Sweden

## Abstract

Background and purpose — Artificial intelligence (AI), deep learning (DL), and machine learning (ML) have become common research fields in orthopedics and medicine in general. Engineers perform much of the work. While they gear the results towards healthcare professionals, the difference in competencies and goals creates challenges for collaboration and knowledge exchange. We aim to provide clinicians with a context and understanding of AI research by facilitating communication between creators, researchers, clinicians, and readers of medical AI and ML research.

Methods and results — We present the common tasks, considerations, and pitfalls (both methodological and ethical) that clinicians will encounter in AI research. We discuss the following topics: labeling, missing data, training, testing, and overfitting. Common performance and outcome measures for various AI and ML tasks are presented, including accuracy, precision, recall, F1 score, Dice score, the area under the curve, and ROC curves. We also discuss ethical considerations in terms of privacy, fairness, autonomy, safety, responsibility, and liability regarding data collecting or sharing.

Interpretation — We have developed guidelines for reporting medical AI research to clinicians in the run-up to a broader consensus process. The proposed guidelines consist of a Clinical Artificial Intelligence Research (CAIR) checklist and specific performance metrics guidelines to present and evaluate research using AI components. Researchers, engineers, clinicians, and other stakeholders can use these proposal guidelines and the CAIR checklist to read, present, and evaluate AI research geared towards a healthcare setting.

Key concepts presented in this review

- Introduction to artificial intelligence (AI) and machine learning (ML) and how these relate to traditional clinical research statistics
- Common pitfalls in AI research
- How to measure and interpret AI and ML performance and how to interpret these measures
- Ethical considerations related to AI and ML in medicine
- Introduction of a Clinical Artificial Intelligence Research (CAIR) checklist, which helps to facilitate understanding, reporting, and interpreting of AI research in medicine.

Machine learning (ML), deep learning (DL), and artificial intelligence (AI) have become increasingly common in orthopedics and other medical fields. Artificial intelligence, defined in 1955, is “the science and engineering of making intelligent machines,” where intelligence is “the ability to learn and perform suitable techniques to solve problems and achieve goals, appropriate to the context in an uncertain, ever-varying world” (Manning 2020).

Machine learning implies models and algorithms that learn from data rather than following explicit rules. Deep learning (DL) is a form of ML that uses large and multilayered artificial neural networks. Neural networks are computational algorithms influenced by biological networks for information processing. They consist of several layers of “neurons” that communicate. By training the neurons how to communicate, interactions develop that solve a particular problem. DL is currently the most successful and general ML approach (Michie et al. 1994, Manning 2020).

Recent technological breakthroughs in computational hardware (like specialized graphics processors [GPUs] and cloud computing), software, and new algorithms have paved the way for a revolution in applications and utility. Together these have resulted in new and exciting developments. Examples range from new drug discoveries (Fleming 2018, Paul et al. 2020) to skin cancer detection (Esteva et al. 2017), automated screening of diabetic retinopathy (Gulshan et al. 2016, 2019), fracture detection in radiographs (Badgeley et al. 2019, Qi et al. 2020, Olczak et al. 2021), detecting rotator cuff tears in MRI (Shim et al. 2020) or vertebral fractures in CT scans (Nicolaes et al. 2019). As many methods require a deeper understanding of computer science, we see engineers perform much of the research geared towards healthcare professionals. This creates challenges between absolute correctness and a technical perspective, and something all stakeholders, including regular clinicians, can understand and benefit from. This paper aims to give clinicians a context and greater understanding of these AI methods and their results.

## Machine learning, deep learning, and artificial intelligence

At its core, AI involves automating complex algorithms, which often depend heavily on statistics. Computation allows for calculations and modeling on a scale that humans could theoretically perform but which are too large and complicated to be feasible.

AI has its own language and different names for concepts familiar to medical professionals. Using different names obfuscates the fact that these are familiar concepts.

In ML, a model is akin to a test. For example, an ML model could test whether a radiograph fulfills the conditions for containing a fracture. Depending on how well the image meets these conditions, it will calculate a probability for a fracture in the image. In contrast, regular statistics investigate individual features’ contribution to a particular outcome, e.g., how much does smoking or alcohol contribute to the risk of having a fracture. The core difference is that AI models generally have a much richer set of features, often in the thousands. Individual features merge into patterns and regularly lose their interpretability. AI models can mostly be considered “black boxes” as the path from input to output is often unclear. The main objective is, therefore, usually predicting a specific outcome.

Another difference from traditional statistics is that many ML models guess the correct answer and improve by studying the errors they make. For example, suppose we present the model with an image. In that case, the model could guess that the probability is 80% that there is a fracture. If we agree that there is a fracture, we can calculate that the error is 20%. By investigating what parameters were not suggesting fracture, we can nudge those parameters into the fracture category to be more likely to predict a fracture the next time. When reporting on AI interventions, the clinical setting is crucial for understanding performance. Clinicians must understand the tasks and performance measures and whether the outcomes are relevant to the clinical setting.

## Classification

A classification task is a task that categorizes observations to a set of known outcomes/classes, for example, type of fracture, normal vs. pathological ECG, or staging of a malignancy. Typically, the model produces a probability score per outcome. When there are 2 outcomes, e.g., fracture or not, we have a dichotomous outcome, a binary classification problem (e.g., “fracture” or “no fracture”). If there are several possible outcomes, e.g., hip fracture Garden 1–4, it is a multi-class classification task.

The model’s core function is to separate the groups, i.e., it is preferred that the network provides probabilities close to 0% and 100% instead of around 50%. For example, an algorithm might state that there is a 20% probability of a fracture. For some purposes, this could be considered sufficient to decide on the absence of pathology, e.g., a suspected type A ankle fracture. In contrast, a scaphoid fracture may be unfortunate to miss, due to the risk of non-union when left untreated. Even at a 20% likelihood of a fracture, we might proceed with an MRI. Therefore, a reliable classifier’s key feature is better separation between groups with few cases in the conflicted region’ in this example, between 20% and 80%.

## Image analysis, segmentation, and localization

Image analysis (also coined as “computer vision”) has gathered much attention and success. It entails analyzing and classifying the contents of images, for example, fractures (Olczak et al. 2021). Sometimes the task is to classify the contents of the image and specify a feature’s location in an image. Suppose the objective is to locate an object in an image, for example, a femur fracture, and mark it with a bounding box (Qi et al. 2020). In that case, the task is image localization or object detection. A similar task is image segmentation. The task is then to mask out regions of interest, for example, marking out the actual boundaries of individual bones or fracture lines.

## Predicting continuous values (regression modeling)

Predicting continuous outcomes is done using regression modeling. Continuous outcomes could be predicting the angle of fracture displacement, the medial clear-space in an ankle radiograph, or the ulna-plus in a wrist radiograph.

## Other tasks

Natural language processing (NLP) deals with language and text management. It could entail translating between languages, interpreting a written journal, generating a journal, or describing an image’s contents.

Clustering is a form of ML where the AI groups data into classes without prior knowledge. For example, given a collection of radiographs, it is given the task of sorting them into groups. For instance, we could let the algorithm find which fractures are similar instead of manually choosing the groups.

## Pitfalls in the classification task for medical data

### Outcome imbalances

Medical data is often skewed, with some outcomes being much more common than others. In general, we are more likely to find a healthy individual than an unhealthy individual. Hence, a negative test for a disease is the most likely outcome. In situations where there are multiple possible outcome types, e.g., an elaborate classification scheme, each outcome becomes less likely. That is, if we have 30 fractures and 3 groups, each subgroup will, on average, contain 10 fractures. In most cases, it becomes even more skewed, as some subgroups are more common than others.

By emphasizing rare cases, giving them more weight in computations during training, we can alleviate the imbalances during training.

### Training and testing

Training is the process of an AI model iterating through a database of cases, with annotations, thereby learning from many examples. One iteration through the entire training set is called an “epoch,” a time-consuming process that can take hours to weeks to reach optimal performance. During this process, it learns the important features of the data.

The testing phase is when the model examines examples it has not seen before and on the basis of which it has to provide an outcome. Predictions of the model are compared with the ground truth in order to evaluate performance.

During testing, model performance may not necessarily reflect accurate performance, as rare cases will be underrepresented. Unfortunately, recruiting more of the underrepresented classes is labor-intensive. For example, suppose we have a class that occurs once in 300 cases. In that case, we need to review at least 600 images to find 2 examples. These rare cases are usually clinically interesting, and the effort needs to be balanced against the clinical importance.

A common practice is to manipulate images at random, e.g., rotating images, to force the network to find features independent of the manipulations applied; this is a form of data augmentation.

### Missing data

Some outcomes are so rare that they will not be present in the data, preventing the algorithm from detecting them. It is a fundamental difference from humans, who can learn what a class looks like before seeing it, e.g., a Pipkin fracture, and recognize when first seeing it.

### Overfitting

An ML model learns by looking at examples. If it learns the training examples too well, it learns the individual patients instead of the problem’s general features, i.e., it learns to recognizes the individual training cases and the expected outcome, rather than the common traits that make up the outcome. Due to the size and flexibility of ML models used, this is a common problem and the reason why the gold standard is to split the data into at least a training and a test set. The test set is kept separate for final evaluation and is not used for training the model and is thus a more objective measure of performance. The same training case, or patient, must not be included in both the training and test set, as this would overestimate the accuracy. A validation set is similar to the test set, but is used to optimize settings for the model during training—and is not always reported. Confusingly, the test set is sometimes called the validation set.

## Performance measures

When reporting on ML algorithms, the clinical setting is essential for understanding the actual performance—and clinicians and readers must understand the results. There are many methods to measure performance, all with their strengths and weaknesses. In Table 1, we present common and widely accepted outcome measures or metrics.

**Table 1.** Evaluation metrics

Measure	Calculation or description
Accuracy	(TP + TN)/(TP + TN + FN + FP)
Sensitivity, true positive rate	TP/(TP + FN)
(TPR), recall	
Specificity	TN/(TN + FP)
Youden’s J	sensitivity + specificity–1
False-positive rate (FPR)	FP/(TN + FP) = 1–specificity
Precision, positive predictive value	TP/(TP + FP)
(PPV)	
Negative predictive value (NPV)	TN/(TN + FN)
F1-score, Dice score	2•precision•sensitivity/
	(precision + sensitivity)
	2•TP/(2•TP + FP + FN)
Model performance curves:	
Receiver operating characteristic	
(ROC) curve	sensitivity (y-axis) against 1–
	specificity (x-axis), i.e.,
	TPR against FPR
Precision-recall (PR) curve	Precision (y-axis) against
	sensitivity (x-axis)
Area under the curve:	
AUC of the ROC curve (AUC)	Statistic of model performance
AUC of the PR-curve (AUPR)	Statistic of model performance
Object detection and localization—image segmentation	
(localization in an image):	
Intersection over union (IoU)	TP/(TP + FP + FN)
Region of interest (ROI)	Used in 2D and 3D image
	segmentation
Continuous data (regression modeling):	
Means squared error (MSE)	∑(true value–prediction)^2^/ number of cases
Root mean squared error (MSE)	√MSE
Mean absolute error (MAE)	∑(true value–prediction)/ number of cases
Text data:	
Bilingual evaluation understudy	Compares generated text with
(BLEU)	reference texts
Recall-oriented Understudy for	Compares generated text with
Gisting Evaluation (ROUGE)	reference texts
Multiple measurements:	
Frequency weighted average	Summarizes many different
	outcomes

TP = true positive, FP = false positive, TN = true negative,

FN = false negative.

### The confusion table

Many of the performance measures presented here are familiar to clinicians from diagnostic testing, e.g., accuracy, specificity, and sensitivity. ML contains a large number of additional performance measures that researchers can report. There is a need to strike a balance between measures that clinicians are familiar with and achieving methodological perfection.

When assessing an experiment’s outcome or a diagnostic test, it is common practice to present outcomes in a confusion table. A binary test has 2 possible outcomes. For a binary diagnostic test (e.g., presence of a fracture in a radiograph or plantar flexion in Simmonds–Thompson’s test for Achilles tendon rupture), we have 4 possible outcomes. Table 2 will serve as the reference for understanding performance measures.

**Table 2.** A 2-by-2 confusion table for a binary test—2 possible outcomes

	Prediction	
Ground truth	Positive (detected)	Negative (not detected)
Positive (disease)	True positive (TP)	False negative (FN)
Negative (normal)	False positive (FP)	True negative (TN)

“Positive” and “negative” do not refer to benefit. Positive (P) refers to a condition’s presence and negative (N) to the condition’s absence.

**Figure 1.**
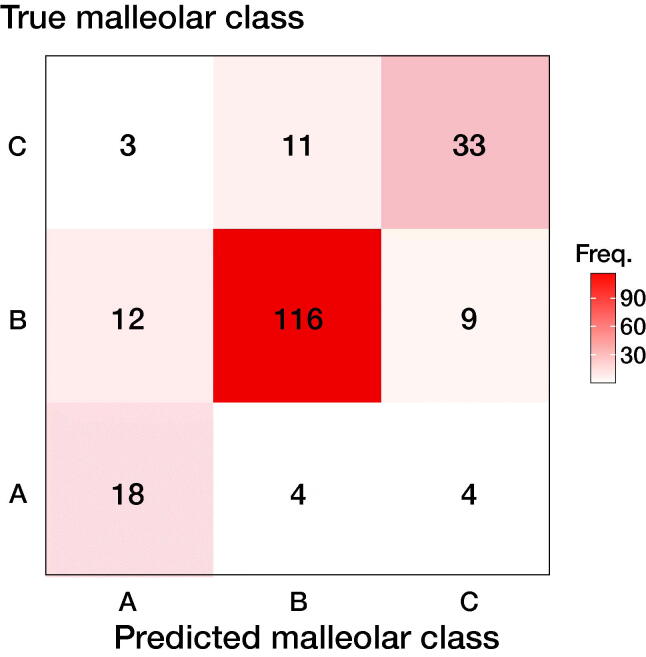
Confusion matrix for an ankle fracture classification experiment, according to Danis-Weber (AO Foundation/Orthopedic Trauma Association (AO/OTA)) classification. There are 26 type A fractures, 137 type B fractures, and 47 type C fractures. Data reproduced from (Olczak et al. 2020).

Suppose we are using a model to classify an ankle fracture into 1 of 3 outcomes—type A, type B, or type C malleolar fracture, excluding the “no fracture” outcome. We present the resulting 3-by-3 confusion table in Figure 1. The usual way to deal with the data is to divide it into subparts, where we look at each outcome separately, as in Table 3.

**Table 3.** Dividing the 3-by-3 confusion matrix from Figure 1 into 3 binary submatrices

		Predicted Type A		Predicted Type B			Predicted Type C	
	True	False		True	False		True	False
True	TP	FN	True	TP	FN	True	TP	FN)
	(18)	(8)		(116)	(21)		(33)	(14)
False	FP	TN	False	FP	TN	False	FP	TN
	(15)	(169)		(15)	(21)		(13)	(150)

There is an underlying decision-making process when an outcome is positive or negative. For example, we might decide that if there is a > 50% chance of the presence of a certain condition, the test is considered positive, and we would get one confusion table. However, if we decided that we need > 90% certainty to decide that a test is positive, we would get less positive test results and fewer false positives (FP). The resulting confusion table would look very different. A screening test might consider a > 20% likelihood as a positive outcome, resulting in many FP but very few false negatives (FN). The threshold where we decide that a test is negative or positive is called the decision threshold or classification threshold.

### Measuring performance

#### Accuracy

Accuracy is defined as the correct classification rate, i.e., the rate of correct findings. For instance, if we had a data set containing 5 fractures in 100 radiographs and all those fractures were detected, we would have 100% accuracy. Suppose we have a test that always indicates “no fracture,” then the performance of the test would be 95% accurate. The second test is very accurate but has no clinical value. Accuracy has limited value for imbalanced data sets.

#### Sensitivity (recall) and specificity

Sensitivity (also known as recall) and specificity are properties well known to clinicians. Sensitivity measures how likely a test is to exclude or detect a condition correctly. We can always achieve 100% sensitivity by saying that everyone has the condition. We would spot every case with the condition, but get also get many false positives.

Specificity represents the true negative (TN) rate, which should usually be high in medical tests, and is balanced with the sensitivity. In a more complex task where we want to differentiate among multiple outcomes, the number of true negatives will dominate for most outcomes, and specificity should generally be high. As with accuracy and other performance measures that consider the TN rate, specificity contains little information of value in unbalanced datasets.

Specificity and sensitivity represent the proportion of TP and TN, respectively, and not the probability of a condition.

A typical use case for high sensitivity is fracture detection. The Ottawa Knee Rule has a sensitivity of 98%, and a negative test will allow us to not go further with further imaging studies. Specificity is roughly 50%, and half will have false-positive tests. Conversely, we care more about specificity in meniscal tears as these are usually less acute, and the bottleneck is often the availability of MRI. Apley’s maneuver has a sensitivity of 20%. However, a specificity of 90% suggests that requesting an MRI will result in few unnecessary exams once encountered. Ideally, a test or algorithm should provide high sensitivity and specificity. However, depending on the clinical setting, we can choose to sacrifice one for the other.

Youden’s J combines specificity and sensitivity into one metric and is a way to summarize them into a single value, ranging from 0 to 1.

#### False positive rate (FPR)

The FPR is the proportion of negative outcomes that have been incorrectly predicted as positive and should be considered the opposite of sensitivity.

#### Positive predictive value (PPV) and negative predictive value (NPV)

Given a prediction, we want to know how likely it is for that prediction to be correct. PPV (also known as precision) answers the question: if we have a set of positive outcomes (cases predicted as positive), what proportion of those outcomes were truly positive? NPV measures the same for negative cases, i.e., if we have a set of negative outcomes, what proportion of those outcomes are genuinely negative?

PPV and NPV, in contrast to specificity and sensitivity, give the probability of an outcome based on the prevalence in the sample.

#### Precision and recall

**Figure 2.**
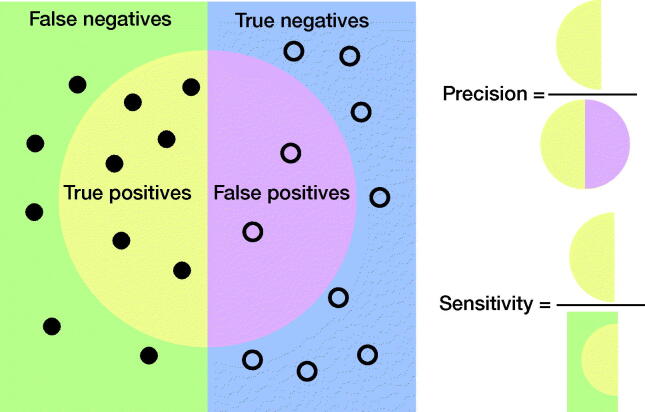
Graphical illustration of precision and sensitivity (or recall). Circles, “●,” represent cases without the disease/class. Bullets, “●,” represent cases with the disease/class.

Precision and recall, as terms, are commonly used in ML studies but relatively unknown in medicine. In epidemiology, precision is the PPV, while recall is the sensitivity. Neither precision nor sensitivity takes into account TNs and, as such, they are less affected by class imbalances in data. Figure 2 illustrates their relationship.

#### F1 score or the Dice score

Class imbalance has become more recognized in medical AI. It has become more common to use performance measures that take class imbalance into account. Precision and sensitivity are less sensitive to class imbalance. The F1 score, or Dice score, is a way to combine precision and sensitivity, and can be understood in terms of data overlap, as in Figure 2. The F1 score is well suited for imbalanced class problems. It is also used in image segmentation and localization tasks; see section “Image segmentation or localization”.

Other good performance measures exist but are not commonly encountered, e.g., the Matthews correlation coefficient (MCC) and other F-scores. See the supplement for details.

### Performance curves and area under the curve (AUC)

We derived the previous performance measures from the confusion table based on classification outcomes. AI classification systems usually yield a probability score as output (e.g., 99% could result in a positive prediction while 3% are in a negative one) and classify data according to a decision threshold. These thresholds are generally arbitrary (e.g., at 50%). However, they can also be tuned on a separate development dataset or derived from the literature. We constructed the confusion table based on whether we detected a condition or not, depending on whether it was present. The outcome of the decision-making process depends on the classification threshold.

To assess the predictions without relying on a single classification threshold, we can compute the negatives’ rate for all thresholds (i.e., from 0 to 1) and plot them in a curve. It is not feasible to compute the confusion matrix and outputs for all possible thresholds. Instead, we compute the confusion matrix for some thresholds, combine them into a curve, and estimate the area under the curve (AUC).

**Figure 3.**
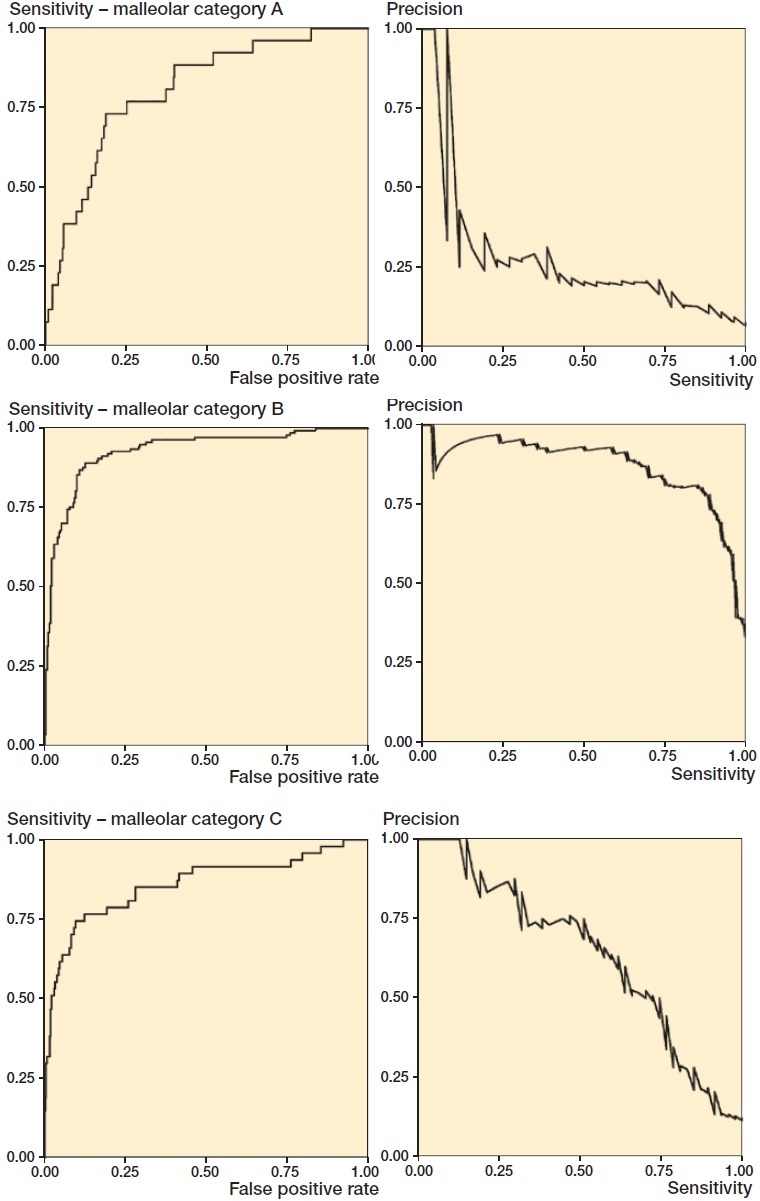
ROC and PR curves for malleolar class predictions. The ROC curves (left) are monotonically growing functions of sensitivity (y-axis) and the FPR (x-axis). The AUC of the ROC curve corresponds to overall model accuracy. The PR-curves (right) have precision on the y-axis and sensitivity on the x-axis. Unlike the ROC, we see that it can oscillate and tends towards zero. The differences between the outcomes are also greater.

By computing AUC, we can estimate generic model performance. The two curves mostly studied are the receiver-operating characteristic (ROC) and precision-recall (PR) curves. In Figure 3, we see the AUC and AUPR curves for the 3 types of malleolar fractures.

#### Receiver operating characteristic (ROC) curve

When research literature mentions AUC, it usually refers to the area under the ROC curve (AUC). We will use AUC for the area under the ROC curve unless otherwise explicitly stated. The ROC curve plots the sensitivity (the y-axis) against the FPR (the x-axis) for all decision thresholds in order to obtain a curve. Computing the area under that curve gives us the AUC, which measures the model’s overall accuracy. The ROC curve’s idea is to measure the model’s ability to separate the groups by penalizing based on how wrong probabilities are.

#### Interpretation

As AUC depends on the specificity, which includes the TN outcomes, it is sensitive to imbalanced data. For a clinical trial or practical application, high AUC risks overestimating performance, because it is related to the accuracy, which is sensitive to data imbalance. One should consider a different performance measure for imbalanced data sets. However, we usually encounter AUC during research and development, where it is used to measure the overall model performance. It does not confine the model to a specific decision threshold, as it is computed over all thresholds. The AUC is well understood, easy to interpret, and has nice properties. See Supplementary material.

#### Precision-recall (PR) curve

Precision is the same as PPV, and recall is the same as sensitivity. The PR curve illustrates the tradeoff between precision and sensitivity and measures the model’s ability to separate between the groups. As neither precision nor sensitivity depends on TN, it is considered well suited to class imbalance data.

Using AUPR to assess a model’s performance, as with the AUC, will measure the model’s performance in a way that is not affected by the classification threshold (Saito and Rehmsmeier 2015). Although it is a valid alternative to AUC, methodological issues with AUPR as a performance measure do exist. There is no clear, intuitive interpretation of AUPR or its properties (unlike AUC, which corresponds to overall accuracy). There is no consensus on what a good AUPR is. AUPR, and similar performance measures, comprise an active research field. However, most of these performance measures still need more research and are not well established. Figure 3 illustrates the differences between AUC and AUPR.

### Image segmentation or localization

Sometimes the research problem is to detect a pathological lesion and locate it in an image to train the model to mark out the areas of interest as a human would. If there is sufficient overlap between the model and human reviewers, it is considered a success. The measures to evaluate segmentation and localization tasks presented next are equally valid for both 2D and 3D data sets.

**Figure 4.**
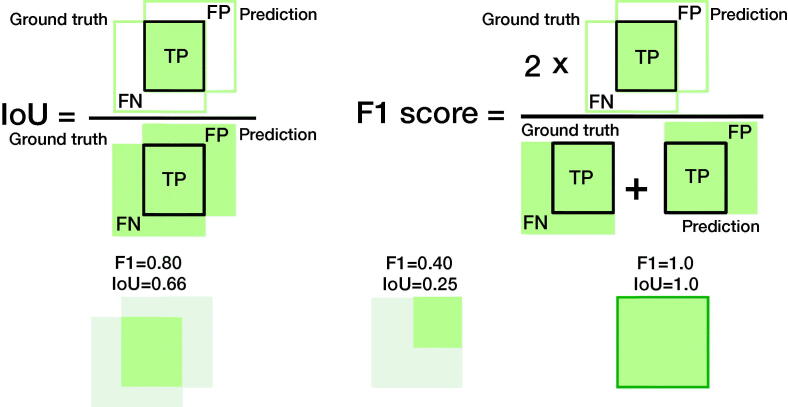
Comparing the IoU and the F1 score in terms of data overlap. The overlapping sets illustrate why both are commonly used performance measures in object detection and image segmentation. The IoU is the percentage of area overlap of correct detection. The F1-score is the “harmonic mean” where the TPs are given additional importance. We can transform one into the other (see supplement). See Table 1 for how to compute IoU and F1 score.

The F1 score is a commonly used performance measure based on its alternate interpretation as overlapping sets; however, the intersection over the union (IoU) is more intuitive (Figure 4).

#### Intersection over union (IoU) or Jaccard index

IoU is used to determine an image segmentation task’s performance in image data, such as radiographs, CT or MRI slices, or pathology slides. It is a measure of the pixel overlap comparing the area of overlap with the combined area of the predicted and actual location (or ground truth) in percentages. While relying on the application and source, it is common to consider > 50% of pixels a sufficient overlap for success.

#### Presence/absence measures

A localization task can also be used to determine the presence or absence of pathology. As this is a type of classification, the same measures used for other classification tasks are suited, e.g., ROC analysis (Chakraborty 2013). However, suppose we are interested in locating a lesion. In that case, the ROC or PR curve cannot measure the model’s ability to locate that region. An alternative measure used to incorporate the localization aspect is the free-response operating characteristic (FROC) (see Supplementary material).

Another option is the region of interest (ROI) analysis, where the image is divided into regions. For example, parts of a brain scan could be divided into their respective cortexes. For each region, the rater assigns a probability that a lesion is located in that region. Plotting the ROC curve, with the number of regions falsely assigned as having a lesion, the performance can then be studied using ordinary ROC analysis. In ordinary ROC analysis, the patient or image is the unit to be observed. In contrast, each region is of interest in ROI (Obuchowski et al. 2000, Bandos and Obuchowski 2018).

### Continuous measurements

Examples of continuous measurements could be estimating the tibiofibular and medial clear spaces in ankle radiographs to assess for syndesmotic injury. As these are continuous values, usually measured in millimeters, an AI model measuring these distances would use regression models to estimate the distance.

#### Root mean squared error (RMSE)

Mean squared error (MSE) is a common performance metric for continuous data. It computes the average squared error between the predicted and actual value. Squaring the error penalizes large errors, and it is thus more sensitive to outliers. Usually, the square root is taken from the MSE, giving the RMSE, which benefits from having the same unit and is easily relatable to the original value.

#### Mean absolute error (MAE)

MAE, or mean absolute deviation, finds the average distance between the predicted and actual value. MAE is less affected by outliers than MSE, as it does not square the difference in values.

### Multiple measurements

Getting an AI model to detect the presence of pathology (2 outcomes) to high accuracy is generally easy, and a trivial task with limited utility. For example, most orthopedic surgeons or radiologists are good at quickly spotting fractures or other pathologies. Rather, use-cases where an AI model will be useful are to classify, locate, or detect many different outcomes or make difficult classifications.

A model will perform differently for each outcome, and we have to take this into account. As the number of outcomes increases, we will have to summarize multiple performance measures for all outcomes. As we would do with a group of individuals where we report a mean, we need to merge multiple outcomes into meaningful summary statistics.

#### Frequency weighted average (FWA)

Taking averages of the individual groups would give excessive importance to small groups. In Figure 2 we noticed that type B fractures were more prevalent than type A fractures and it makes sense that they should contribute more to the overall accuracy. Weighting according to frequency (FWA), excluding true negatives when they are very dominant, can be written as:

$$\mathrm{FWA}=\frac{\sum_{{{}_{case}}_{=\text{ 1}}}^{{}_{last}}n_{case}\cdot \;measure_{case}}{\sum_{{{}_{case}}_{=\text{ 1}}}^{{}_{last}}n_{case}}$$

where n is the number of cases. For example, frequency weighted average AUC (from Figure 1) would become:

AUC_FWA_ = (24 · 0.8 + 137 · 0.93 + 47 · 0.86)/(24 + 137 + 47) = 0.90.

FWA can be applied to any metric, for instance, AUPR_FWA_ = (24 · 0.27 + 137 · 0.87 + 47 · 0.63)/(24 + 137 + 47) = 0.75.

#### Medical language generation

Medical language generation involves the generation of medical text (e.g., diagnostic text or discharge summaries), with or without the use of input (e.g., radiographs). For example, Gale et al. (2018) trained a system to produce descriptive sentences to clarify deep learning classifiers’ decisions when detecting hip fractures from frontal pelvic radiographs.

The most common word-overlap measures in medical text generation are BLEU (Papineni et al. 2002) and ROUGE (Lin 2004). BLEU measures content overlap between the model and ground truth texts and penalizes short generated captions using a brevity penalty. BLEU-1 considers single words, while BLEU-2, -3, -4 consider texts with 2 to 4 words, respectively.

ROUGE-L(Recall) is in biomedical captioning the most common ROUGE variant. It measures the ratio of the length of the longest word subsequence in the human-generated text shared and the system-generated text. The measure complements BLEU by focusing on the human-generated text’s length instead of the system-generated text.

We also note that various language generation evaluation measures exist, such as METEOR (Banerjee and Lavie 2005), CIDEr (Vedantam et al. 2015), and SPICE (Anderson et al. 2016). It is important to remember that human language complexity is vast and cannot be captured fully by these measures. Human evaluation of text is therefore commonly required as a supplement.

## Ethical considerations and methodological biases

New technology comes with new ethical dilemmas, and AI is no exception. The potential benefits of AI are real, as are ethical considerations. As we invest resources in research and then the software, hardware, and other logistics, resources come from elsewhere. The ramifications of AI are considerable, but clinicians are poorly informed (Felländer-Tsai 2020). We briefly describe common ethical dilemmas that clinicians should be aware of and take into account. The fundamental ethical principles that concern medical practice and patient care and treatment comprise beneficence, non-maleficence, respect for patient autonomy, and justice.

### Data and privacy

ML and AI are powerful methods often described as “data-hungry,” as they are needed to learn desired patterns and capture rare or unusual cases. AI models, at their core, conclude statistical relationships and therefore thrive on large amounts of data during training, which encourages large-scale data collection. Data, even in the right hands, can constitute a risk to patient integrity. For example, oversharing, overuse of personal data, or data theft all constitute risks to patient privacy and risk the data falling into the wrong hands or being used for the wrong purposes. Medical data is sensitive and cannot always be shared, causing problems for reproducibility and reporting on models’ outcomes. However, there are ways to anonymize and share data legally and responsibly (Hedlund et al. 2020), and this is highly encouraged.

### Bias and fairness

Bias in AI mainly originates from the input data and the development process, and the design decision. These biases transfer to the output data, and an AI model will learn the data’s prejudice (Mittelstadt et al. 2016). Clinicians, biased by the AI interpretation, risk perpetuating that bias. Commonly acknowledged biases and confounders are gender, socioeconomic, and race. For example, a skin cancer detector trained on a dataset dominated by fair skin can have problems detecting melanoma in dark-skinned patients (Adamson and Smith 2018, Kamulegeya et al. 2019). Badgeley et al. (2019) successfully predicted hip fractures from radiographs. However, when they compensated for socioeconomic and logistical factors and healthcare process data (e.g., different scanners), model performance fell to random.

Bias comes from the source and handling of data as well as the design choices during algorithm creation. Above all, it is important to recognize, examine, and reflect on AI studies’ biases (Beil et al. 2019).

### Informed consent and autonomy

AI poses a risk to patient autonomy and integrity. When AI models produce difficult-to-explain outcomes based on unknown data, it becomes difficult to base decisions on their output. AI models also pose a risk to clinician autonomy. As AI systems become more prevalent, there is a risk that society will divert the responsibility for decision-making to algorithms that are incompletely understood. Clinicians and healthcare systems might implicitly become forced to implement and follow them against better judgment, which will also implicitly force patients to subject themselves to AI (Lupton 2018).

### Safety and interpretability

The power of AI systems comes from their ability to use large amounts of data to create complex models that consider thousands of parameters. However, AI models, as developed today, are difficult to understand and interpret. AI models are mostly “black boxes.” What happens inside the model is usually unknowable. However, other medical technology and even many human analyses can also be considered black boxes. It is impossible to back-track the process fully in practice.

Understanding ML models is an active field of research. One way to address the challenge is to learn to create interpretable models from the start (Rudin 2019).

One popular way to understand AI models is to visualize the activating regions, i.e., the regions that lead to the classification decision get mapped in vivid colors. These can be called heat, saliency, or class activation maps. Another method is to produce bounding boxes that constitute the region of interest. However, whether the correct or incorrect region is displayed, they still do not explain why the model reacted to that region (Rudin 2019). Such auxiliary maps can capture some AI mispredictions, but far from all. Other methods to achieve interpretability include showing similar reference cases or deriving uncertainty measures (Pocevičiūtė et al. 2020).

Transparency in AI is crucial for actual clinical implementations where errors could have critical implications. To critically assess AI results in the clinical workflow, we could supply standardized “model facts labels” along with the AI tool (Sendak et al. 2020); this is similar to the facts labels accompanying drugs to inform practitioners on suitable usage. Transparency could also help compensate for the sensitive nature of the data used to train and test them, which usually cannot be shared.

### Responsibility and liability

Who is responsible and liable for AI interventions is not always clear. A model that is 95% accurate is wrong 5% of the time. It is common for an AI model that is excellent at a task to fail at examples obvious to a human observer. Some errors are within normal parameters. If the patient accepted the AI intervention, we might consider this an unfortunate but acceptable risk. However, if an AI model suggests a course of action, but the underlying rationale is not clear, clinicians might not follow it. Suppose the recommendation was correct, and not following them caused harm to the patient. Are clinicians responsible? Suppose they followed the AI recommendation, and it turned out to be a critical error, constituting malpractice. Who is liable and responsible, legally but also morally? Currently, most AI interventions are tools that assist clinicians, rather than replacing them, and then the physician remains responsible.

## Proposed guidelines for evaluating and presenting AI/ML research

**Figure 5.**
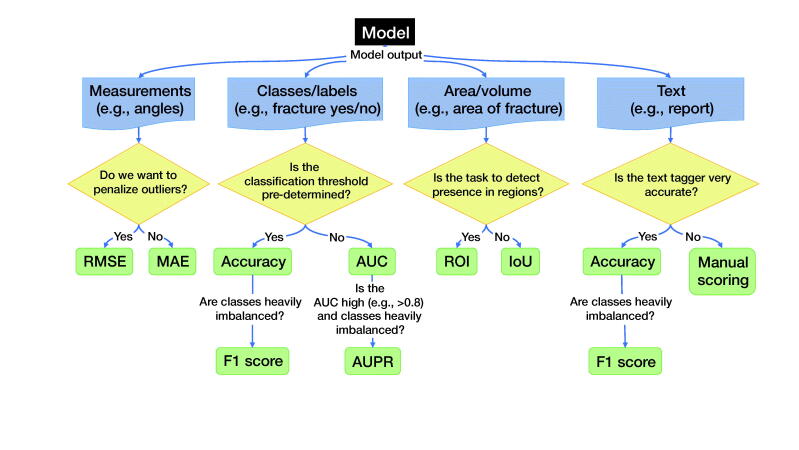
Recommendations for choosing outcome metrics suitable for clinicians. The selected measures are selected for their (1) suitability and (2) their interpretability to a clinician. Deviations from these are possible; however, they need to be motivated, and we recommend also reporting these metrics. IoU (Intersection over Union); ROI (Region of Interest); MAE (Mean Average Error); RMSE (Root Mean Squared Error); AUC (Area Under the Receiver Operating Characteristic curve; AUPR (Area Under the Precision-Recall curve).

Based on the previous discussion, we propose guidelines and a checklist for reporting and presenting AI and ML to clinicians and other non-machine learning experts. We first state our recommendations (Figure 5) on reporting and presenting AI and ML research to clinicians and provide a checklist for reporting (Table 4).

**Table 4.** Clinical AI Research (CAIR) Checklist Proposal

Section	Reporting recommendations
**TITLE AND ABSTRACT**	
*** Include that the method contains or uses an AI/ML.*** Broad terms such as “artificial intelligence” or “machine learning” are encouraged, but “deep learning” or similarly broadly specific terms would also work well. More precise terms are best suited in the abstract.	
*** State the AI tool’s intended use or purpose,*** in a disease context in the title and/or the abstract. What is the targeted condition?	
**INTRODUCTION**	
The introduction should focus on the clinical problem. The AI component is the tool used to solve the clinical problem. If possible, explain the AI’s intended part within the clinical pathway.	
**METHODS**	
*** State inclusion and exclusion criteria, at the participant and the input data level, separately.***	
State why these criteria were used.	
How were rare pathologies handled?	
*** Describe how the input data was acquired, selected,****and handled,* and include any form of preprocessing before analysis. If there were some specific considerations in handling the data, this should also be specified.	
Was the data split into separate train, validation, and test sets?	
Are there any differences in how test and training sets were selected and processed?	
How were patients or cases that occur more than once handled? Can they be found in both the test and training set? For example, same patient at different points in time or duplicate data.	
Are positive and negative cases from different sources? (For example, perhaps different machines are used in high- or low-probability settings, and the algorithm learns this pattern instead?)	
If there were minimum requirements on the data, state what those requirements were.	
*** Specify if there was a human–AI interaction handling input data and level of expertise of the people handling it.***	
How was the ground truth established (e.g., double review with consensus, consensus review, single review, secondary sources)?	
What was the level of expertise of the source or reviewers? What level of noise was present (e.g., Cohen’s kappa).	
If there was training involved in handling the data, this should be specified.	
*** Describe how missing or poor-quality data was handled.***	
Were extreme values or outliers handled separately? Explain how and why.	
*** State the AI model’s specifications, design, and the parameters used in training it.***	
The model’s data requirements, to serve its purpose, need to be clearly stated (e.g., data format, dimensions, time, etc.).	
How was the data preprocessed? It should be stated separately for training and test sets.	
What was the model architecture? Was a pre-trained model used? Was it pre-trained for the current study?	
If it was a pre-trained model, is the data the model was pre-trained on also part of the current data sets?	
What regularizers were used? (For example, dropout, white noise, batch normalization, stochastic weight averaging, etc.)	
How was the loss calculated? If a non-standard loss function was used, why was this particular loss chosen?	
What model-specific parameters were used in training the model? For example, learning rate, number of epochs, etc.	
*** State the specific version of the AI model used in the study.*** AI models are likely to undergo many iterations. It is important for reproducibility and tracking changes in the model if reused or implemented in a later study.	
*** Specify the output of the AI. The output affects the model interpretation and post-processing.***	
What was the type of output? For example, probabilities, bounding boxes, text, segmented images, models?	
*** Explain how the output contributed to decision-making and evaluation of the model.***	
In what way was the output decided upon? Sometimes the reason for deciding on that output needs to be specified. For example, when the output depends on a decision threshold, and the model used non-standard thresholds, or when different thresholds are used for different outcomes, it might be necessary to explain why they are different.	
If the output was used in later steps, how was it used? Include explanations of how the outputs informed, or led to, subsequent steps. For example, was the output used in subsequent steps by a user to inform an action or was it combined with a different model?	
*** How was outcome performance measured?*** At times it could be necessary to state why a performance measure was chosen over a different performance measure.	
The performance measure most likely familiar to the clinicians should be the primary reporting measure. Sometimes, alternate or additional measures are required but it is important to ensure that these are adequately explained.	
In the statistical section, specify the exact version of the measure used, e.g., ROUGE-L-Recall, Rouge-L-Precision, or Rouge-L-F1.	
How was confidence evaluated? Bootstrapping, Monte Carlo simulation, p-value?	
For suggestions on how to choose performance measures, see Figure 5.	
**RESULTS**	
*** Describe the results of analysis and performance errors.*** If no such analysis was performed, justify why not. Performance errors and failure analysis are important for AI models and help communicate the limitations of the model.	
**DISCUSSION AND OTHER INFORMATION**	
*** State if and how the AI model/data can be accessed, including any restrictions to access or reuse. If it is not possible, state why.*** Include any details and license. While this is highly desirable, it is not always possible to make data or models readily available or make them available online.	
*** Describe ethical considerations and implications of the model, and/or research.*** Biases and limitations, the input data or output, that impact generalizability should also be considered.	

Guidelines for publishing, reviewing, and evaluating reporting of AI and ML content to clinicians. Clinical trials and clinical trial protocols, including AI interventions, should adhere to the CONSORT-AI (Liu et al. 2020a, 2020b) and SPIRIT-AI (Rivera et al. 2020) checklists.

However, those contain minimal reporting requirements. Besides, most studies are not in a clinical trial stage, and some of those recommendations are not necessarily applicable. The table elaborates on some important parts of reporting on studies utilizing AI/ML components.

### Recommendations for reporting outcomes

Figure 5 comprises recommendations for choosing outcome metrics suitable for clinicians. We choose these measures as they are (1) suitable and, in general, (2) most interpretable to a clinician. While the discussion regarding what makes a good choice is still ongoing, and deviations from our suggested metrics are possible, we expect that our suggestions will assist the indecisive clinician. We recommend including these metrics alongside any other metrics.

#### Continuous

*Classification.* AUC is a standard measure that most clinicians are familiar with or have at least encountered. It is, though, ill-suited for heavily imbalanced data sets, where AUPR should accompany the AUC measure. If the performance in AUC is low, the additional information from AUPR is less relevant.

*Measurements.* For continuous variables, e.g., angles, coordinates, or VAS pain, we can use root mean squared error (RMSE) or mean absolute error (MAE). Both translate to values interpretable on the original scale and are familiar to many clinicians from traditional statistics.

Historically, we have been more interested in RMSE as outliers tend to be a major concern. For example, after wrist fracture surgery, most patients will have low VAS-pain levels. We are then primarily interested in identifying failures that risk high levels of VAS. Machine learning allows for new applications and, under some circumstances, we will at times prefer the MAE. For example, if the system draws a bounding box around a fracture, the box must be close to the fracture site most of the time. If the objective is to enhance efficiency while quickly viewing images, we are less concerned with rare, complex fracture cases. These will, regardless of the bounding box, require more attention.

#### Area or volumes

The F1 score is a common performance measure for segmentation performance in images. However, we argue that its interpretation is non-intuitive compared with the IoU, as shown in Figure 4. We therefore recommend using IoU. As an alternative, used in particular for 3D imaging, we recommend using ROI, which is more intuitive than most alternate performance measures.

#### Medical text

If we compare to a known text, such as in biomedical image captioning, we can use BLEU and ROUGE-L (Kougia et al. 2019). However, we observe that these 2 measures do not assess clinical correctness (Table 5). A single word could change the meaning of the text, for example, changing “presence” to “absence,” or adding “no” to a sentence, and could potentially cause adverse outcomes for patients. A human review will be necessary to ascertain clinical correctness. If we had a very accurate clinical tagger (i.e., tagging text with clinical keywords), we would estimate clinical correctness via accuracy or F1 score, e.g., by tagging both the generated and the reference clinical texts and measuring accuracy and F1 over the extracted tags.

**Table 5.** Example sentences for medical text analysis using BLEU and ROUGE

GT	Subtle impacted intertrochanteric hip fracture	B1	B2	B3	B4	ROU
H1	No subtle impacted intertrochanteric hip fracture	83.3	81.6	79.4	76.0	100.0
H2	There is a hip fracture clearly appearent on the radiograph	20.0	14.9	28.1	38.6	40.0

H1 scored higher than H2 compared with the ground truth (GT, human-generated), using BLEU-1/-2/-3/-4 (B1, B2, B3, B4) and ROUGE-L (ROU). However, given the ground truth (GT), H2 is clinically correct, while H1 is not.

#### Accuracy

Accuracy is an easily understood and often requested performance measure. Even its weakness, overestimating performance, is easy to understand. If the data is heavily imbalanced, however, the F1 score is the preferred choice.

*Clinical Artificial Intelligence Research (CAIR) Checklist*

See Table 4.

## Discussion

AI and ML will most likely impact medicine in more ways than we can imagine. Arguing that clinicians need to be involved, we began by describing different tasks and pitfalls in machine learning and shared some ways to address them.

We followed by presenting the related concept of performance measures. Performance measures describe the result of the study. Using the right performance measure will give a correct context to the outcome. However, performance measure choice is not always clear and occasionally depends on an experiment’s stage and the audience. For a prospective study or development of an AI model, a measure such as AUC is appropriate. When we use an AI model as an intervention in a clinical trial or in a production setting, where we implement a specific AI system, the actual expected performance is more important. MCC or precision-recall analysis with AUPR and F1-score are more suitable, as AUC could overestimate the model’s performance (Chicco and Jurman 2020).

We discussed some of the fundamental ethical problems and consequences of algorithmic medicine and AI interventions. We believe that this is essential for understanding and evaluating AI studies, including their limitations. Ethical considerations can limit individual AI systems, but those limitations are sometimes necessary to safeguard the patients, who are the ultimate beneficiaries of medical AI.

The Enhancing the Quality and Transparency of Health Research, EQUATOR (Pandis and Fedorowicz 2011), network defines an AI intervention as an intervention that relies on an AI/DL/ML component (Liu et al. 2020a, 2020b, Rivera et al. 2020). In line with the growing importance of AI research in healthcare, the SPIRIT (Standard Protocol Items: Recommendations for Interventional Trials) 2013 and the CONSORT (CONsolidated Standard for Reporting Trials) 2010 were amended in 2020 with SPIRIT-AI and CONSORT-AI checklists. SPIRIT-AI and CONSORT-AI are additional checklists meant to deal with the particulars of AI studies. In particular, they address the particular biases involved. They do not specify how to conduct AI studies but give minimal recommendations for reporting on them. Similar protocols for other study types are under development. For diagnostic and prognostic studies, STARD-AI (Standards for Reporting Diagnostic Accuracy-Artificial Intelligence) reporting guidelines are in development. Moreover, TRIPOD-ML (Transparent Reporting of a Multivariable Prediction Model for Individual Prognosis or Diagnosis–Machine Learning) is in development (Liu et al. 2020b). However, CONSORT-AI and SPIRIT-AI are minimal reporting checklists.

We presented a proposal for recommendations and guidelines on reporting AI and ML research to clinicians and other healthcare stakeholders. We also proposed the CAIR checklist to facilitate these recommendations. We envision this proposal as the starting point of a broader consensus process on reporting, presenting, and understanding AI studies’ outcomes. We also hope to help healthcare professionals and other healthcare stakeholders interpret these studies.

We have not fully covered all aspects of AI and ML in medicine or orthopedics, which would be an impossible task. This paper focuses on 3 important areas for understanding and evaluating AI research in medicine. We have picked tasks commonly found in medical AI studies that are most likely to be encountered in orthopedics research. The selection can, and will, change as the field and clinicians’ familiarity with it evolve. For example, while drafting this paper, the CONSORT-AI and SPIRIT-AI guidelines were published, but TRIPOD-ML and STARD-AI have not yet been.

## Conclusion

With the advancement of technology, computational power, and a great deal of research, AI will be an important clinical tool. For some this is a cause of concern, while for others this is an opportunity to improve health outcomes. What matters, in the end, is what is best for the patient. New tools can help clinicians do a better and more reliable job and automate tedious and trivial tasks, allowing them to focus on complex tasks.

There are also risks associated with AI. The risk is that clinicians do not understand and take part in the process around them. Alternatively, they may embrace and not understand what the implications are. If low quality or wrongly guided research dominates, the implementation of meaningful outcomes might suffer. Ethical considerations that clinicians face every day are not always shared or understood by the developers behind the tools, who could have a different agenda than clinicians.

While we are very far from a time when AI will replace clinicians, we are in a time when clinicians must deal with and benefit from AI. Clinicians need to understand the changes, research, and results that are happening every day. To guide those developments, what is most needed is for clinicians to be part of this development. In order to do that, they need to understand it. The goal of the CAIR checklist is to facilitate this.

## Ethics, funding, and potential conflicts of interest

MG is supported by grants provided by Region Stockholm (ALF project) and JO by grants provided by the Karolinska Institute. MG is a co-founder and shareholder in DeepMed AB. CL is an employee and shareholder of Sectra AB.

## Supplementary Material

Supplemental Material
