# Supplementary material for: Presenting artificial intelligence, deep learning, and machine learning studies to clinicians and healthcare stakeholders: an introductory reference with a guideline and a Clinical AI Research (CAIR) checklist proposal
Source: Acta Orthop. 2021 May 14;92(5):513–25. doi: 10.1080/17453674.2021.1918389 (PMC8519529; doi:10.1080/17453674.2021.1918389)
Supplement: Supplemental Material [file IORT_A_1918389_SM4147.pdf]

# Supplementary data

### *Area under receiver operating characteristic curve (AUC) heuristic*

Table S1. presents classification thresholds for interpreting the AUC. These are widely used heuristics, based on empirical observations and are not mathematically derived.

**Table S1. Commonly used accuracy classifications for AUC (Wang et al. 2010).**

| AUC range | Classification |
|---|---|
| 0.9 < AUC ≤ 1.0 | Excellent |
| 0.8 < AUC ≤ 0.9 | Good |
| 0.7 < AUC ≤ 0.8 | Not good |
| 0.6 < AUC ≤ 0.7 | Worthless |
| 0.5 ≤ AUC ≤ 0.6 | Random |

### *F-scores*

Precision and sensitivity (also known as recall) are less sensitive to class imbalance. The F-score is a way to combine precision and sensitivity. Depending on the application and what we are interested in, we can modify this score to penalize different errors, i.e., precision or sensitivity, or FN or FP.

The general F-score, $F_\beta$, is defined as

$$F_\beta = (1 + \beta^2)\,\frac{(precision \bullet sensitivity)}{(\beta^2 \bullet precision + sensitivity)}$$

and attaches  times more importance to precision than sensitivity.

The most commonly encountered F-score is the F1-score (with ß = 1), also known as the Dice score. F1 is the harmonic mean of precision and sensitivity and considers FN and FP errors as equally costly.

The F1 score is well suited for imbalanced class problems and used in image segmentation and localization tasks.

Any ß is possible but two common are F2 and F0.5. F2 considers precision more important and F0.5 considers sensitivity more important.

### *The relationship between Intersection over Union (IoU) and F1-score*

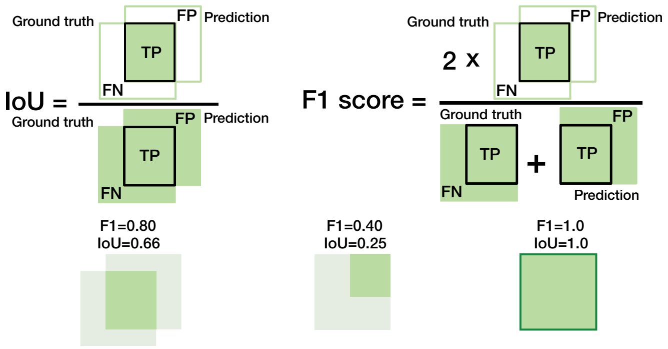


Figure S1. The IoU and the F1 and their graphical definitions.

IoU is a more conservative performance than F1 and IoU ≤F1. From Table 1 we can see that the IoU can be written as

$$IOU = \frac{TP}{TP + FP + FN} = \frac{|ground\ truth\ true \cap predicted\ true|}{|ground\ truth\ true| + |predicted\ true| - |ground\ truth\ true \cap predicted\ true|}$$

and the F1 score as

$$F_1 = \frac{2TP}{2TP + FP + FN} = \frac{2 \bullet |ground\ truth\ true \cap predicted\ true|}{|ground\ truth\ true| + |predicted\ true|}$$

.

We can convert from IoU to F1, and back, via the relationships

$$F_1 = \frac{2 \bullet IoU}{1 + IoU}$$

and

$$IOU = \frac{F_1}{2 - F_1}$$

## Matthews correlation coefficient (MCC)

As we described, class imbalanced problems, where one outcome dominates, are common in medicine. The resulting imbalanced data is difficult for performance measures to capture fairly.

MCC considers the confusion table and computes the correlation between the observed outputs and the classifier's predictions. It is a discrete version of the Pearson correlation coefficient for two outcomes. It balances the entire confusion matrix and is therefore also suited for imbalanced problems (Boughorbel et al. 2017, Chicco and Jurman 2020). Many consider MCC the optimal performance measure for binary classification. But it, and its properties are not easy to understand, and MCC is not widely used.

$$MCC = \frac{(TP \cdot TN - FP \cdot FN)}{\sqrt{(TP + FP)(TP + FN)(TN + FP)(TN + FN)}}$$

## Free-response operating characteristic (FROC)

In FROC analysis the rater is given the task of listing all abnormal areas with a suspected lesion and rate the probability that there is a lesion. The proportion of correctly located (within some distance) and classified abnormalities are plotted on the y-axis and the x-axis is the average number of FP per patient (Obuchowski et al. 2000). An alternative approach, called the alternative FROC (AFROC) is to use the probability of at least 1 false positive finding on the x-axis. AFROC allows for computing the AUC as a summary measure of model accuracy (Chakraborty and Winter 1990, Obuchowski et al. 2000, Bandos et al. 2009, Chakraborty 2013, Hillis et al. 2017).

Compared to ROI which gives the probability for a lesion in a region, FROC can be used to estimate the number of lesions in a region, and the individual probabilities for each lesion.

**Bandos A I, Rockette H E, Song T, Gur D.** Area under the Free-Response ROC Curve (FROC) and a related summary index. Biometrics 2009; 65(1): 247-56.

**Boughorbel S, Jarray F, El-Anbari M.** Optimal classifier for imbalanced data using Matthews Correlation Coefficient metric. PLOS ONE 2017; 12(6): e0177678.

**Chakraborty D P.** A brief history of free-response receiver operating characteristic paradigm data analysis. Academic Radiology 2013; 20(7): 915-9.

**Chakraborty D P, Winter L H.** Free-response methodology: alternate analysis and a new observer-performance experiment. Radiology 1990; 174(3 Pt 1): 873–81.

**Chicco D, Jurman G.** The advantages of the Matthews correlation coefficient (MCC) over F1 score and accuracy in binary classification evaluation. BMC Genomics 2020; 21(1): 6.

**Hillis S L, Chakraborty D P, Orton C G.** ROC or FROC? It depends on the research question. Medical Physics 2017; 44(5): 1603-6.

**Obuchowski N A, Lieber M L, Powell K A.** Data analysis for detection and localization of multiple abnormalities with application to mammography. Academic Radiology 2000; 7(7): 516-25.

**Wang N, Zeng N N, Zhu W.** Sensitivity, specificity, accuracy, associated confidence interval and ROC analysis with practical SAS implementations. Northeast SAS User Group proceedings, Section of Health Care and Life Sciences, Baltimore, Maryland, 14-17 November 2010, 1-9.
